# Der prognostische Stellenwert der Körperzusammensetzung („body composition“) in der onkologischen Viszeralchirurgie

**DOI:** 10.1007/s00104-024-02189-5

**Published:** 2024-10-29

**Authors:** Saleem Elhabash, Nils Langhammer, Ulrich Klaus Fetzner, Jan-Robert Kröger, Ioannis Dimopoulos, Nehara Begum, Jan Borggrefe, Berthold Gerdes, Alexey Surov

**Affiliations:** 1 Klinik für Allgemein‑, Viszeral‑, Thorax- und Endokrine Chirurgie, Universitätsklinikum Minden, Ruhr-Universität Bochum, Hans-Nolte-Str. 1, 32429 Minden, Deutschland; 2 Universitätsinstitut für Radiologie, Neuroradiologie und Nuklearmedizin, Universitätsklinikum Minden, Ruhr-Universität Bochum, Hans-Nolte-Str. 1, 32429 Minden, Deutschland

**Keywords:** Körperzusammensetzung, Sarkopenie, Myosteatose, Viszerales Fettgewebe, Computertomographie, Body composition, Sarcopenia, Myosteatosis, Visceral fat tissue, Computed tomography

## Abstract

Das Screening des Ernährungsstatus spielt eine entscheidende Rolle im perioperativen Management von Krebspatienten und ist im Rahmen der Zertifizierungsvorgaben von Krebszentren durch die Deutsche Krebsgesellschaft (DKG) vorgeschrieben. Die verfügbaren Screening-Tools unterscheiden nicht zwischen Muskel- und Fettgewebe. Die Fortschritte der Computertomographie (CT) und der Magnetresonanztomographie (MRT) wie auch die automatisierte PACS(„picture archiving communication system“)-getriggerte Bildanalyse erlauben in den letzten Jahren erstmals eine detaillierte Analyse des Fettgewebes und der Muskelqualität in der klinischen Routine. Es gibt eine rasch zunehmende Evidenz dafür, dass die Parameter der Körperzusammensetzung („body composition“), insbesondere die reduzierte Muskelmasse, mit einem negativen Einfluss auf das „overall survival“, die Chemotherapietoxizität und chirurgische Komplikationen bei Tumorpatienten assoziiert sind. In diesem Artikel fassen wir die aktuelle Literatur zusammen und stellen damit den klinischen Einfluss der „body composition“ bei viszeralonkologischen Erkrankungen dar.

## Abstract (en)

Screening of nutritional status of cancer patients plays a crucial role in the perioperative management and is mandatory for the certification of oncological centers by the German Cancer Society (DKG). The available screening tools do not differentiate between muscle and adipose tissue. Recent advances in computed tomography (CT) and magnetic resonance imaging (MRI) as well as the automatic picture archiving communication system (PACS) imaging analysis by high performance reconstruction systems have recently enabled a detailed analysis of adipose tissue and muscle quality. Rapidly growing evidence shows that body composition parameters, especially reduced muscle mass, are associated with adverse outcomes in cancer patients and have been reported to negatively affect overall survival (OS), disease-free survival (DFS), toxicity associated with chemotherapy and surgical complications. In this article, we summarize the recent literature and present the clinical influence of body composition in oncological visceral diseases.

## Hintergrund

Die Behandlungsqualität in der Viszeralchirurgie verbessert sich stetig. Dies kann auf ein sorgfältiges perioperatives Management, neue minimal-invasive Techniken und die Verbesserung des Screenings auf relevante Komorbiditäten zurückgeführt werden. Insbesondere die Komorbiditäten spielen für perioperative Komplikationen und das postoperative Outcome eine entscheidende Rolle [1–6].

Intra- und postoperative Komplikationen haben eine signifikante negative Auswirkung auf das „overall survival“ (OS) sowie das „disease-free survival“ (DFS) oder das rezidivfreie Überleben (RFS) bei onkologischen Patienten. Risikofaktoren wie Malnutrition, schlechter ASA(American Society of Anesthesiologists)-Status, erhöhter BMI (Body-Mass-Index), chirurgische Notfalleingriffe, erhöhter intraoperativer Blutverlust, verlängerte Operationszeit, intraoperative Komplikationen und Kontamination chirurgischer Wunden sind Faktoren, die mit postoperativen Komplikationen assoziiert sind [7–12].

Um diese Risiken zu minimieren und somit auch die Qualität der chirurgischen Ergebnisse zu verbessern, wurden sowohl eine umfassende perioperative Diagnostik als auch Konzepte der Prähabilitation implementiert, um eine raschere Erholung der Patienten nach großen chirurgischen Eingriffen zu ermöglichen (Enhanced-recovery-after-surgery[ERAS]-Konzept). Die Evaluation prognostischer Risikofaktoren, wie beispielsweise der Ernährungsstatus und Performance-Status (Karnofsky, Eastern Cooperative Oncology Group etc.), hämatologische Grundlagen (Albumin, Hämoglobin) sowie multimodale therapeutische Programmen wie Schmerztherapie, Stressreduktion, frühzeitige enterale Ernährung und Mobilisation sind integraler Bestandteil dieses Konzepts [13].

Das Screening des Ernährungsstatus von Krebspatienten durch validierte Tools wird vor allem in Krebszentren genutzt und ist im Rahmen der Zertifizierungsvorgaben dieser Zentren durch die Deutsche Krebsgesellschaft (DKG) vorgeschrieben. Die verfügbaren Screening-Tools basieren zumeist auf der BMI-Bestimmung, dem tumorbedingten Gewichtsverlust und anthropometrischer Messungen der Muskelmasse [14]. Ein potenzielles Problem dieser Tools ist, dass der BMI zwar mit dem Gesamtfettanteil des Körpers korreliert, dabei jedoch weder den Unterschied zwischen Muskel- und Fettgewebe beachtet noch die Menge des Fettgewebes und des Fettdepots, die sich mit zunehmender Körpergröße dementsprechend erhöhen [15]. So wird beispielsweise bei Patienten mit kolorektalem Karzinom sowohl bei niedrigem BMI als auch bei hohem BMI von erhöhter Mortalität berichtet [16]. Dem übereinstimmend zeigte sich, dass die Messungen der Sarkopenie in der Computertomographie (CT) und Magnetresonanztomographie (MRT) im Vergleich zum BMI deutlich validere prädiktive Parameter für die prognostische Bestimmung des anthropomorphischen Zustands sind [15].

Es gibt eine rasch zunehmende Evidenz dafür, dass die Parameter der Körperzusammensetzung, insbesondere die reduzierte Muskelmasse, mit negativem Outcome bei Tumorpatienten assoziiert sind [17]. Aktuelle Berichte zeigen, dass eine verringerte Quantität (Sarkopenie) oder Qualität der Muskulatur (Myosteatose) Parameter zur Vorhersage des OS, der Chemotherapietoxizität und chirurgischer Komplikationen bei Patienten mit unterschiedlichen Tumoren sind [18, 19].

Die starke Evidenz, dass Sarkopenie als prognostischer Marker bei onkologischen Patienten genutzt werden kann, hat auch im Bereich der Viszeralchirurgie weitere Forschungsarbeiten angestoßen. Es gibt starke Hinweise, dass die Modifikation der „body composition“ eine zu onkologischen Interventionen ähnliche Bedeutung für das Behandlungsergebnis besitzen kann [17–23].

Es finden sich widersprüchliche Darstellungen bezüglich des prognostischen Wertes von Übergewicht (hohe viszerales und/oder subkutanes Fettgewebe) in der Vorhersage des OS bei Karzinompatienten [22].

In diesem Artikel fassen wir die aktuelle evidente Literatur basierend auf großen Metaanalysen zusammen und legen damit den klinischen Einfluss der „body composition“ bei viszeral-onkologischen Erkrankungen dar.

## Parameter der „body composition“ und Analysemethodik

**Abb. 1:**
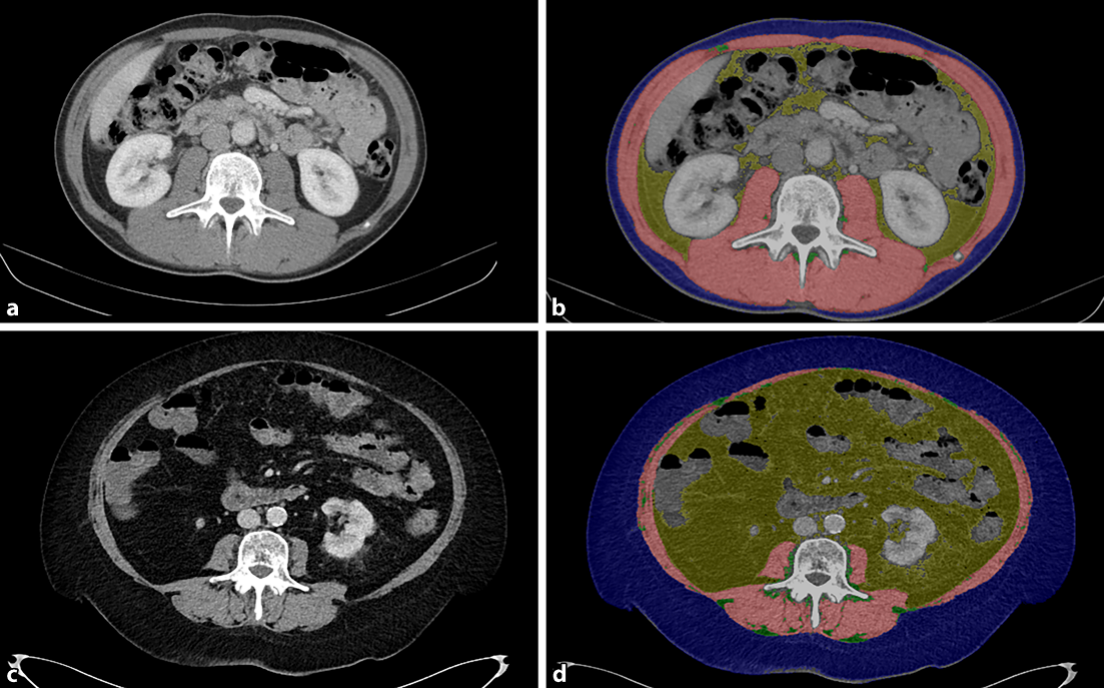
Darstellung der CT-graphisch vermessenen Körperzusammensetzung, Muskelfläche (*rot*), Fläche des viszeralen Fettgewebes (*gelb*), Fläche des subkutanen Fettgewebes (*blau*), Fläche des intramuskulären Fettgewebes (*dunkelgrün*). Patient mit normaler Muskelmasse und geringem viszeralem Fett (**a**), segmentierte Areale der Körperzusammensetzung (**b**), Patient mit reduzierter Muskelmasse und deutlicher viszeraler Adipositas (**c**), segmentierte Areale der Körperzusammensetzung (**d**)

Die Fortschritte der Computertomographie und der Magnetresonanztomographie wie auch die automatisierte PACS-getriggerte Bildanalyse durch Hochleistungsrekonstruktionsrechner erlauben in den letzten Jahren erstmals eine detaillierte Analyse der Körperkonstitution („body composition“) in der klinischen Routine. Aufgrund ihres Standards im Staging und Follow-up vieler Tumorpatienten wird die Abdomen-CT letztlich am häufigsten dafür genutzt [24]. Die meistgenutzte Messmethode ist die Quantifizierung der „body composition“ in der axialen Ansicht auf Höhe L3 (Abb. 1)**.**

Die folgenden Parameter der Körperzusammensetzung können aus der Schnittbildgebung extrahiert werden:

- Fläche der Skelettmuskulatur,
- Fläche des viszeralen Fettgewebes (VF),
- Fläche des subkutanen Fettgewebes (SF) und
- Fläche des intramuskulären Fettgewebes (IF).

Aus der Skelettmuskelfläche und der Körpergröße kann der sogenannte Skelettmuskelindex (SMI) kalkuliert werden, (SMI = Muskelfläche [cm^2^]/Körpergröße [m]^2^; [19, 25]).

Die zumeist genutzten Cut-off-Werte für die Parameter der Körperzusammensetzung sind in Tab. 1 summiert (Tab. 1).

**Tab. 1:** Die meistgenutzten Grenzwerte für die Parameter der „body composition“

Parameter	Männer	Frauen
Skelettmuskulatur	52,4 cm^2^/m^2^	38,6 cm^2^/m^2^
Skelettmuskulatur	BMI < 25 kg/m^2^: 43 cm^2^/m^2^ BMI >= 25 kg/m^2^: 53,0 cm^2^/m^2^	41 cm^2^/m^2^
Viszerales Fettgewebe	100 cm^2^	
Subkutanes Fettgewebe	100 cm^2^	
Muskeldichte	41 HU für Patienten mit einem BMI < 25; 33 HU für Patienten mit einem BMI von ≥ 25	

*BMI* Body-Mass-Index, *HU* Hounsfield-Einheit

Durch künstliche Intelligenz (KI) können alle Abmessungen bereits vollautomatisch vorgenommen werden.

## Der prognostische Stellenwert der Körperzusammensetzung („body composition“) in der onkologischen Viszeralchirurgie

### Ösophagus- und Magenkarzinome

Sarkopenie ist ein starker und unabhängiger Parameter für das schlechtere Gesamtüberleben in dieser Patientengruppe (Tab. 2, [a]).

**Tab. 2:** Einfluss der Parameter der Körperzusammensetzung auf das klinische Outcome bei Magen‑, Ösophaguskarzinom und Adenokarzinom des ösophagogastralen Überganges (AEG)

**(a) Gesamtüberleben**										
*Parameter der Körperzusammensetzung*	*Diagnose*	*Behandlung*	*Gesamtzahl einbezogener Studien (n)*	*Gesamtzahl Patienten (n)*	*Univariable Analyse*			*Multivariable Analyse*		
					*HR*	*95* *%-KI*	*p‑Wert*	*HR*	*95* *%-KI*	*p‑Wert*
*Viszerale Fettareale *[22]	Magenkarzinom	Operation alleinig/mit Chemotherapie	5	2769	1,28	1,09–1,49	0,002	**–**	**–**	**–**
*Sarkopenie *[32]	Magenkarzinom	Operation	10	3865	1,9	1,68–2,12	0,00001	2,02	1,71–2,38	0,00001
*Sarkopenie *[33]	Ösophaguskarzinom	Operation	18	3413	1,6	1,25–1,95	0,0001	–	–	–
*Myosteatose *[34]	Magenkarzinom	Operation alleinig/mit Chemotherapie	12	5904	1,46	1,32–161	< 0,001			
**(b) Rezidivfreies Überleben**										
*Sarkopenie* [33]	Ösophaguskarzinom	Operation	18	3413	1,73	1,04–2,87	0,03	–	–	–
*Sarkopenie *[32]	Magenkarzinom	Operation	14	4242	–	–	–	1,97	1,71–2,26	0,00001
*Viszerale Fettareale* [35]	Magen‑, Ösophaguskarzinom, AEG	Operation alleinig/mit Radiochemotherapie	8	3646	0,90	0,72–1,14	0,02	–	–	–
**(c) Auftreten schwerer postoperativer Komplikationen (Clavien-Dindo** **>** **Grad 3), univariable Analyse**										
*Parameter der Körperzusammensetzung*	*Diagnose*	*Behandlung*	*Gesamtzahl einbezogener Studien (n)*		*Gesamtzahl Patienten (n)*		*OR*		*95* *%-KI*	*p‑Wert*
*Sarkopenie* [32]	Magenkarzinom	Operation	7		2912		1,54		1,03–2,24	0,04
*Sarkopenie *[33]	Ösophaguskarzinom	Operation	5		1072		1,31		0,96–1,80	0,09
*Viszerale Fettareale *[35]	Ösophagus- und Magenkarzinom	Operation alleinig/mit adjuvanter oder neoadjuvanter Chemotherapie	5		2503		1,42		0,97–2,09	0,08

Hohes viszerales Fettgewebe und Myosteatose sind auch wesentliche Prädiktoren für schlechteres Gesamtüberleben bei Patienten mit Ösophagus- und/oder Magenkarzinom (Tab. 2, [a]). Allerdings liegen keine evidenzbasierten Daten dazu im multivariablen Setting vor.

Sarkopenie ist auch ein unabhängiger Faktor für ein geringeres rezidivfreies Überleben bei Patienten mit Magenkarzinom (Tab. 2, [b]).

Laut Literatur spielt das viszerale Fettgewebe keine prädiktive Rolle für das rezidivfreie Überleben in dieser Gruppe (Tab. 2, [b]).

Sarkopenie ist mit dem Auftreten schwerer postoperativer Komplikationen bei Magenkarzinomen vergesellschaftet (Tab. 2, [c]). Sie hat allerdings keine prognostische Bedeutung beim Ösophaguskarzinom (Tab. 2, [c]).

### Pankreaskarzinom

Bei Patienten mit kurativer Therapie eines Pankreaskarzinoms ist die Sarkopenie ein starker und unabhängiger Prädiktor des reduzierten Gesamtüberlebens und rezidivfreien Überlebens (Tab. 3, [a und b]). Das Vorliegen einer Myosteatose führt auch zur Reduktion des Gesamtüberlebens (Tab. 3, [a, univariable Analyse]).

**Tab. 3:** Einfluss der Parameter der Körperzusammensetzung auf das klinische Outcome bei Pankreaskarzinom und periampullärem Karzinom

**(a) Gesamtüberleben**										
*Parameter der Körperzusammensetzung*	*Diagnose*	*Behandlung*	*Gesamtzahl einbezogener Studien (n)*	*Gesamtzahl Patienten (n)*	*Univariable Analyse*			*Multivariable Analyse*		
					*HR*	*95* *%-KI*	*p‑Wert*	*HR*	*95* *%-KI*	*p‑Wert*
*Sarkopenie *[36]	Pankreaskarzinom	Operation alleinig/mit adjuvanter oder neoadjuvanter Chemotherapie	7	1885	1,8	1,41–2,28	0,00001	1,62	1,27–2,07	0,0001
*Myosteatose* [24]	Periampulläres Karzinom/Pankreaskarzinom	Operation mit Chemotherapie oder alleinige Chemotherapie bei Stadium IV	8	1413	1,93	1,60–2,33	< 0,0001		**–**	
**(b) Rezidivfreies Überleben**										
*Sarkopenie *[36]	Operation alleinig/mit adjuvanter oder neoadjuvanter Chemotherapie	7 (univariabel)/8 (multivariabel)	1885 (univariabel)/2065 (multivariabel)	1,7	1,29–2,24	0,0002	1,86	1,34–2,6	0,0002	
**(c) Auftreten schwerer postoperativer Komplikationen (Clavien-Dindo** **>** **Grad 3), univariable Analyse**										
*Parameter der Körperzusammensetzung*	*Behandlung*		*Gesamtzahl einbezogener Studien (n)*		*Gesamtzahl Patienten (n)*		*OR*	*95* *%-KI*		*p‑Wert*
*Sarkopenie* [36]	Operation alleinig/mit adjuvanter oder neoadjuvanter Chemotherapie		8		2065		1,06	0,77–1,47		0,71

Sarkopenie hat allerdings keinen relevanten Einfluss auf das Auftreten postoperativer Komplikationen (Tab. 3, [c]).

Es liegen derzeit keine evidenten Daten bezüglich der prognostischen Rolle des Fettgewebes in der viszeralen operativen Therapie des Pankreaskarzinoms vor.

### Kolorektales Karzinom

Sarkopenie ist ein starker und unabhängiger Parameter, welcher mit dem schlechteren Gesamtüberleben bei Patienten mit kurativ operierten kolorektalen Karzinomen assoziiert ist (Tab. 4, [a]). Myosteatose spielt ebenso eine prädiktive Rolle, allerdings liegen keine Daten aus multivariablen Analysen dazu vor (Tab. 4, [b]).

**Tab. 4:** Einfluss der Parameter der Körperzusammensetzung auf das klinische Outcome bei kolorektalem Karzinom

**(a) Gesamtüberleben**																						
*Parameter der Körperzusammensetzung*		*Behandlung*			*Gesamtzahl einbezogener Studien (n)*			*Gesamtzahl/Patienten (n)*	*Univariable Analyse*							*Multivariable Analyse*						
									*HR*		*95* *%-KI*		*p‑Wert*			*HR*		*95* *%-KI*			*p‑Wert*	
*Sarkopenie *[37]		Operation			10			3865	1,9		1,68–2,12		0,00001			2,02		1,71–2,38			0,00001	
*Myosteatose *[24]		Operation mit Chemotherapie oder alleinige Chemotherapie bei Stadium IV			11			9614	1,70		1,49–1,94		0,01			–						
**(b) Rezidivfreies Überleben**																						
*Parameter der Körperzusammensetzung*				*Behandlung*		*Gesamtzahl einbezogener Studien (n)*				*Gesamtzahl/Patienten (n)*				*Univariable Analyse*								
														*HR*	*95* *%-KI*					*p‑Wert*		
*Myosteatose *[38]				Nicht spezifiziert		7				8572				1,00	0,95–1,05					0,17		
*Sarkopenie *[37]				Operation		25				15.446				1,55	1,29–1,88					0,00001		
**(c) Auftreten postoperativer Komplikationen, univariable Analyse**																						
*Parameter der Körperzusammensetzung*	*Behandlung*		*Komplikation*				*Gesamtzahl einbezogener Studien (n)*					*Gesamtzahl Patienten (n)*					*OR*		*95* *%-KI*			*p‑Wert*
*Sarkopenie *[37]	Operation		Schwere postoperative Komplikationen (Clavien-Dindo > Grad 3)				16					4514					1,72		1,1–2,68			0,02
*Viszerale Fettareale *[39]	Nicht spezifiziert		Anastomoseninsuffizienz				3					526					2,4		1,06–5,44			0,04
			Wundinfektionen (SSI)				4					659					3,22		1,95–5,32			< 0,00001
			Overall-Morbidität				4					659					2,33		1,56–3,48			< 0,0001

Auch hat die Sarkopenie einen signifikanten Einfluss auf das rezidivfreie Überleben laut univariabler Statistik (Tab. 4, [b]).

Sarkopenie und hohes viszerales Fettgewebe beeinflussen das Auftreten schwerer postoperativer Komplikationen (univariable Analysen; Tab. 4, [c]).

### Tumoren der Leber und Gallenwege

Sowohl bei Patienten mit hepatozellulärem Karzinom (HCC) als auch bei Patienten mit cholangiozellulärem Karzinom (CCC) ist Sarkopenie ein starker und unabhängiger Prädiktor des reduzierten Gesamtüberlebens und rezidivfreien Überlebens (Tab. 5, [a und b]). Myosteatose hat einen starken Einfluss auf das Gesamtüberleben bei HCC. Allerdings liegen nur Daten univariabler Analysen vor (Tab. 5, [a]).

**Tab. 5:** Einfluss der Parameter der Körperzusammensetzung auf das klinische Outcome bei hepatozellulärem Karzinom (HCC) und cholangiozellulärem Karzinom (CCC)

**(a) Gesamtüberleben**										
*Parameter der Körperzusammensetzung*	*Diagnose*	*Behandlung*	*Gesamtzahl einbezogener Studien (n)*	*Gesamtzahl/Patienten (n)*	*Univariable Analyse*			*Multivariable Analyse*		
					*HR*	*95* *%-KI*	*p‑Wert*	*HR*	*95* *%-KI*	*p‑Wert*
*Sarkopenie *[40]	CCC	Operation	13	2359	2,0	1,47–2,73	0,01	2,26	1,75–2,26	0,00001
*Sarkopenie *[29]	HCC	Operation	7	1795	2,0	1,56–2,44	0,00001	2,17	1,48–3,19	0,0001
*Myosteatose *[24]	HCC	Operation mit Chemotherapie	3	2004	1,88	1,4–2,52	0,07	–		
**(b) Rezidivfreies Überleben**										
*Sarkopenie *[40]	CCC	Operation	13(univariabel)/13 (multivariabel)	2359 (univariabel)/1881(multivariabel)	1,89	1,12–3,17	0,02	2,2	1,75–2,75	0,00001
*Sarkopenie *[29]	HCC	Operation	7/6	1795 (univariabel)/1538 (multivariabel)	1,85	1,44–2,37	0,00001	1,79	1,28–2,5	0,0006
**(c) Auftreten schwerer postoperativer Komplikationen (Clavien-Dindo** **>** **Grad 3), univariable Analyse**										
*Parameter der Körperzusammensetzung*	*Diagnose*	*Behandlung*	*Gesamtzahl einbezogener Studien (n)*			*Gesamtzahl Patienten (n)*		*OR*	*95* *%-KI*	*p‑Wert*
*Sarkopenie* [40]	CCC	Operation	10			1612		1,23	1,07–1,41	0,004

Sarkopenie ist stark assoziiert mit dem Auftreten schwerer postoperativer Komplikationen bei Patienten mit CCC (Tab. 5, [c]). Diese Daten sind allerdings nur im univariablen Setting getestet.

Es liegen bis dato keine evidenten Daten bezüglich der prognostischen Rolle des Fettgewebes bei HCC und CCC in der viszeralen Chirurgie vor.

## Diskussion

Die vorliegenden Daten belegen die prognostische Rolle unterschiedlicher Parameter der „body composition“ in der onkologischen Viszeralchirurgie.

In der onkologischen Chirurgie entwickelt sich die Prärehabilitation basierend auf dem präoperativen Risikoprofil stetig. Sie hat die Zielsetzung, modifizierbare Risikofaktoren in der kurzen präoperativen Phase umzukehren, um die physiologische Reserve der Patienten zu aktivieren und zu verbessern. Bislang sind keine Parameter der „body composition“ in die ERAS-Evaluation integriert [13, 26].

Verschiedene klinische Studien sind aktuell dabei, den Effekt der Prärehabilitation auf den Therapieerfolg bei der Behandlung von Krebspatienten darzulegen und zeigen dabei vorerst eine vorläufige Evidenz, dass die multimodale Prärehabilitation vorteilhaft für Sarkopeniepatienten ist. Dabei wird die funktionale Kapazität verbessert und insbesondere eine raschere Erholung von chirurgischen Eingriffen erzielt [27, 28].

In der vergangenen Dekade war die Bestimmung der „body composition“ in der Schnittbildgebung im Fokus vieler wissenschaftlicher Arbeiten und steht nun aufgrund neuer automatisierter Arbeitsabläufe mit und ohne KI als klinischer Parameter zur Verfügung. Dabei besteht eine starke Evidenz, dass die quantitativen Messwerte der „body composition“ als prognostische Marker bei Krebserkrankungen sehr vielversprechend sind [29]. Zusätzlich weisen die aktuellen Guidelines der European Working Group of Sarcopenia in Older People (EWGSOP) darauf hin, dass nicht nur die Muskelquantität dabei wichtig ist, sondern vor allem auch die Muskelqualität, beides Parameter die in der CT- und MRT-Bildgebung routinemäßig untersucht werden können [19]. Patienten mit ähnlicher Muskelmasse, aber unterschiedlichem Anteil intramuskulärer Fetteinlagerungen haben eine reduzierte Muskelkraft und dadurch eine höhere Rate an Gebrechlichkeit („frailty“) und funktioneller Einschränkung, die sich wiederum als kritische Prognosefaktoren bei onkologischen Patienten darstellen [25, 30]. Dies führte zu einem gesteigerten Interesse an unterschiedlichen Muskelparametern und deren klinischer Assoziation im onkologischen Setting. Wie für die Sarkopenie zeigen hier eine Vielzahl von Studien und Metaanalysen, dass die Myosteatose ein pathologisches Phänomen darstellt, das mit einem schlechteren OS und DFS bei unterschiedlichen Krebsentitäten sowie mit einem Anstieg der Inzidenz der postoperativen Komplikationen assoziiert ist [18].

Die Zusammenhänge zwischen der „body composition“ und dem relevanten klinischen Outcome in der onkologischen Viszeralchirurgie sind multifaktoriell. Die Relevanz der Sarkopenie und Myosteatose für den Verlauf chirurgischer Eingriffe und onkologischer Therapien kann sowohl durch metabolische wie auch mechanische Dysfunktion des Skelettmuskels erklärt werden [19]. Eine der wichtigsten Auswirkungen der Sarkopenie und Myosteatose ist die metabolische Dysfunktion. Diese steht in Zusammenhang mit einer Insulinresistenz durch die Beeinträchtigung des Insulin-Signaling-Pathway und die Veränderungen in der oxidativen Kapazität des Muskels [19]. Das könnte zu einer systemischen Inflammation und oxidativem Stress führen, welcher die Proteinsynthese, den Umsatz der Muskelproteine und die Funktionalität der inneren Organe verhindern könnte [19]. Als Konsequenz daraus wurde die Hypothese aufgestellt, dass diese metabolischen und mechanischen Dysfunktionen die Krebsentwicklung und das Fortschreiten aktiver Krebserkrankungen beeinflussen könnten.

Weiterhin kann der bei der Myosteatose beobachtete Umbau von Typ-II-Muskelfasern in Typ-I-Muskelfasern möglicherweise die Muskelkontraktion beeinträchtigen und dadurch eine mechanische Dysfunktion durch Verschlechterung der Mobilität und Funktion erzeugen [19].

Viszerales Fettgewebe verfügt ebenso über eine systemische Wirkung, die über zahlreiche metabolisch aktive Substanzen vermittelt wird [31]. Dazu gehören proinflammatorische Zytokine wie Tumornekrosefaktor‑α (TNF-α), Interleukin 1 und Interleukin 6 [31]. Neben der Aktivierung der systemischen Inflammation können diese auch Tumorwachstum und Metastasierung begünstigen [31].

Die vorliegende Arbeit zeigt auch deutliche Limitationen der aktuellen Literatur auf. Während die Zusammenhänge zwischen der Sarkopenie und dem klinischen Outcome bei allen onkologischen Erkrankungen analysiert wurden, liegen bis dato keine ausreichenden Ergebnisse bezüglich der prognostischen Rolle der Myosteatose und/oder des Fettgewebes vor. Dies soll bei den zukünftigen Studien und Metaanalysen berücksichtigt werden.

## Fazit

- Präoperative klinische Evaluationsmaßnahmen werden zunehmend interessanter zur Optimierung der Behandlung von Patienten unter Kombinationstherapien mit Operation und adjuvantem Therapieregime in der viszeralen Onkologie.
- Viele Studien zeigen, dass einzelne Parameter der Körperzusammensetzung unabhängige prädiktive Faktoren für ein schlechteres Outcome sind. Daher wird die Hinzunahme dieser Parameter in die präoperativen Evaluationstools empfohlen. Das könnte möglicherweise zu einer verbesserten Identifikation aussagefähiger Prognosefaktoren und hierdurch zu der Entwicklung eines individualisierten Patientenmanagements mit verbessertem chirurgischem Outcome beitragen.
- Die Messungen der Parameter sind mit der Computertomographie am kosteneffektivsten, da diese die routinemäßig eingesetzte Diagnostik im Tumorstaging darstellt.
- In der Literaturrecherche besteht ein mangelhafter Konsens bezüglich der Messmethoden für die VFA, Myosteatose und Sarkopenie. Darüber hinaus fehlen einheitliche diagnostische Cut-off-Werte [24]. Weiterhin wurden die meisten Studien an Patientenkohorten aus Asien durchgeführt und nur wenige Studien existieren mit Populationen aus Europa und Nordamerika.
- Daher sind weitere Untersuchungen notwendig, um die Mechanismen und prognostischen Effekte der in der Schnittbildgebung gemessenen Körperkonstitutionsparameter zu verstehen. Es ist des Weiteren sinnvoll, deren Relevanz in Bezug auf verschiedene Krebsentitäten und geschlechtsspezifische Merkmale aufzuschlüsseln. Zusätzlich sind prospektive Studien notwendig, um die Rolle der Prärehabilitation im präoperativen Management unter Beachtung der „body composition“ weiter auszuloten.

## Abkürzungen

BMI: Body-Mass-Index
CT: Computertomographie
DFS: Disease free survival
DKG: Deutsche Krebsgesellschaft
ECOG: Eastern Cooperative Oncology Group
ERAS: Enhanced recovery after surgery
KI: Künstliche Intelligenz
MRT: Magnetresonanztomographie
OS: Overall survival
PACS: Picture archiving communication system
RFS: Recurrence-free survival
